# Expression of concern: ‘MALAT1 promotes gastric adenocarcinoma through the MALAT1/miR-181a-5p/AKT3 axis’ (2019), by Lu *et al.*

**DOI:** 10.1098/rsob.230303

**Published:** 2023-08-31

**Authors:** Open Biology editorial team

Following the publication of ‘MALAT1 promotes gastric adenocarcinoma through the MALAT1/miR-181a-5p/AKT3 axis’, the Editorial team have concerns regarding the validity of the data used in this study.

We are issuing an expression of concern here and investigating this as a matter of urgency; we will notify readers as to the results of our investigation as soon as possible.

